# Environmental concentrations, characteristics and risk assessment of microplastics in water and sediment along the Western Cape coastline, South Africa

**DOI:** 10.1016/j.heliyon.2023.e18559

**Published:** 2023-07-22

**Authors:** Danielle Julius, Adetunji Awe, Conrad Sparks

**Affiliations:** Department of Conservation and Marine Sciences, Cape Peninsula University of Technology, Cape Town, South Africa

**Keywords:** Microplastics, Coastal waters, Sediment, Polymers, Risk assessment

## Abstract

Plastic debris is accumulating in all environments globally and South Africa’s poor waste management plan has led to an increase in plastic contamination throughout the country. Information about microplastics (MPs) in urban and rural coastal environments in South Africa is poor. The aim of this study was to determine coastal MP concentrations in water (particles/L) and sediment (particles/kg). Sampling took place in summer of 2020 during low tide at 14 sites, along the coast of the Western Cape, South Africa. MPs were extracted and analysed based on shape, color, size and polymer type (using an ATR-FTIR). An ecological risk assessment was done to assess the potential risks posed by MPs in all sample types. Sediment MP concentrations (185.07 ± 15.25 standard error particles/kg) were higher than water (1.33 ± 0.15 particles/L). Gordon’s Bay (site 12) had the highest MP concentrations in sediment samples (360 ± 36.74 particles/kg), identifying harbors as the main source for MP contamination. Kalk Bay (site 9) displayed the highest concentration in water samples (4.97 ± 0.18 particles/L), suggesting that the source of MPs are from stormwater outfall pipes and human activities. Filaments were the most dominant MP shape (89%) for all samples, with black/grey (water) and transparent (sediment) being the most dominant colors (31% and 31% respectively). Dominant sizes were 1000–2000 μm in water and 2000–5000 μm in sediment. Polyethylene terephthalate (PET) (29%) was the most dominant polymer type recorded in water samples and natural fibres (mainly cotton) (32%) recorded in sediment. Based on the risk assessment, MPs recorded at Mouille Point (site 6) poses the greatest ecological risk associated with polymers. MP concentrations reported in this study provide a baseline for future studies along the Western Cape coastline of South Africa.

## 1 Introduction

As much as 8 million tonnes of plastic end up in the marine environment and make up 80% of all marine debris from surface waters to deep-sea sediments [1]. Due to its lightweight and durability, plastic debris accumulates in all environmental compartments, from beaches and surface ocean waters, deep sea and sediments, arctic ice, freshwater systems, soil and terrestrial niches, to indoor environments as well as food and drinking [2]. Between 60%–80% of anthropogenic debris in marine environment is in the form of plastic [[3], [4], [5]]. Plastic debris is being released into the marine environment at an alarming rate, raising global concerns regarding the health of the environment [[6], [7], [8]].

Microplastics (MPs) are defined as plastic particles smaller than 5 mm and are classified as primary MPs and secondary MPs [6]. MP types vary and are characterised based on shape (i.e fragments, filaments, films and spheres) polymer type, size and even color. MPs enter the marine environment in various ways and is an emerging marine contaminant and potential pollutant [7]. The wide range of properties (size, shape, density) MPs possess influences its distribution in the marine environment i.e sink or float. In addition, MP properties change over time as a result of degradation [7,9]. The problem is that plastic products have a long durability and often outlive their utility, becoming waste and enter the marine environment.

MPs can stem from land- and sea-based sources. Land-based sources include urban and stormwater runoff, sewer overflows, littering and illegal dumping and trading, inadequate waste management, industrial activities, tyre abrasion and construction. Sea-based sources include activities from the fishing industry, oil drilling, rivers, aquaculture and maritime activities [6,[10], [11], [12]]. Studies have identified microfibres as being the most predominant type of secondary MPs [[13], [14], [15]] and is linked to domestic waste and sewage-sludge disposal sites [13]. Other studies have identified harbours and marinas as potential sources of MP contamination due to harbour dredging, accidental discharge of oil and chemical spills, shipping paint and repair works from boating maintenance, uncontrolled disposal and leakage of industrial and urban waste [16].

Sources of MP contamination and plastic composition are responsible for the spatial distribution of MPs in coastal areas of South Africa. South Africa’s coastline stretches approximately 3400 km and comprises natural bays, coves, estuaries, harbours and marinas [17]. Factors contributing to the prevalence of MPs are urbanisation and industrialisation along South Africa’s coastline. Plastic concentrations at sea-surfaces along the coastal shelf of KwaZulu-Natal in South Africa are significantly higher near urbanised areas [15]. Other studies investigated plastic debris on South Africa’s beaches and found that higher concentrations of plastics are found at beaches close to urban-industrial areas [18]. MPs in the Port of Durban were significantly high within in the harbour, particularly at sites closer to stormwater outfalls [19].

There are various environmental factors and sources that contributes towards the potential contamination of MPs along the coastline of the Western Cape of South Africa. Sources include a combination of land- and marine based sources from river systems, streams, stormwater and sewage outfalls, wastewater treatment works (WWTWs), dredging and dumping and shipping activity [[20], [21], [22]]. Between 60 and 90% of plastic waste is land-based and is stranded along the coastline [20]. One of the major contributors towards urban litter entering the sea is run-off from stormwater drainage systems. Stormwater systems transport large amounts of rainwater from the streets and have the potential to move waste from gardens, roofs, footpaths, streets and parking lots. In South Africa, stormwater run-off is not processed before it is discharged into the marine environment and there are approximately 124 authorised outfall pipes in the Western Cape [23]. To date, there are very few studies that have reported on the concentrations and risks posed by MPs in the Western Cape. MP concentrations (mainly filaments) and ecological risks in coastal sediment of a marina in Simon’s Town (False Bay, Cape Town) were highest close to a stormwater outfall pipe, with this site posing the highest ecological risk for rocky shores close to the site sampled [24].

Risks associated with MP contamination are potential indicators of the impact of MPs. A hazard ranking model of plastic polymers has been developed to incorporate the chemical hazard of additives, monomers, polymers and polymerization [25]. This hazard ranking model can be used to assess the hazardous effects of plastic polymers on human health and the environment. The hazard ranking model is used to conduct a risk assessment associated with MPs present in the environment.

Given the lack of knowledge about MPs in the Western Cape of South Africa, the aim of this study was to measure the abundances, characteristics and potential risks of MPs in water and sediment within the Western Cape of South Africa. The objectives of the study were to: 1) measure the concentrations of MPs in coastal water and sediment at 14 sites (urban and rural) in the Western Cape, 2) record the shape, color, size and polymer type of MPs in the Western Cape, and 3) determine the environmental risks of MPs in the Western Cape, South Africa.

## 2 Materials and methods

### 2.1 Study area

**Fig. 1:**
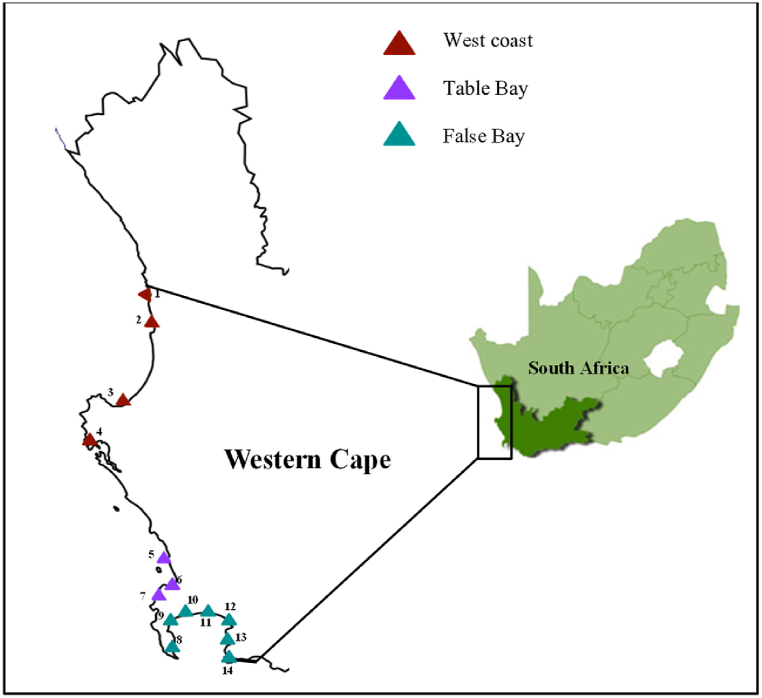
Study site including sampling stations 1: Lambert’s Bay, 2: Eland’s Bay, 3: Velddrif, 4: Saldanha Bay, 5: Bloubergstrand, 6: Mouille Point, 7: Maiden’s Cove, 8: Simon’s Town, 9: Kalk Bay, 10: Strandfontein, 11: Strand, 12: Gordan’s Bay, 13: Rooi Els and 14: Pringle Bay.

The Western Cape has a narrow continental shelf with a coastline stretching over 1000 km. The coastal environment is diverse, dynamic and influenced by both the Benguela Current to the west and Agulhas Current to the south (Fig. 1). For this study, the coastline is divided into three regions: West Coast (rural), Table Bay (urban) and False Bay (mainly urban).

The West Coast is located within the southern Benguela upwelling system. The Benguela current is a slow flowing current and water circulation is driven by large-scale winds, resulting in an anticyclonic motion and thermohaline forcing [26]. Longshore equatorial winds result in coastal upwelling [27]. The northern boundary of the West Coast is Lambert’s Bay, approximately 270 km north of Cape Town and includes coastal towns Langebaan, Saldanha Bay, Velddrif and Paternoster. River systems that are potential sources of MP contamination include the Berg River, Verlorenvlei and Jakkalsrivier [23]. The West Coast is a mixed-use area, where fishing, aquaculture and agriculture are the dominant activities. In addition to these activities, sea-side resorts and rural towns (in particular, informal settlements in this region) have made the West Coast susceptible MP contamination.

To the south of the West Coast region is the Table Bay region, also located within the southern Benguela upwelling system. Water circulation within the bay is primarily driven by wind, shelf width and gradient and offshore currents and tides [28]. During summer, south-easterly winds cause currents to flow northwards in an anti-clockwise direction within in the bay [27]. Surface currents are generally weak in this area, with very little to no influence from outside currents [28]. The coastline stretches 19 km from Melkbosstrand to Cape Town. The coastline consists of 13 km of sandy beach (between Blouberg and Table Bay harbour), 3 km of rocky shore (between Blouberg and Mouille Point) and 4 km of artificial shore protection and breakwaters at the Port of Cape Town. There are several river systems and streams that are potential sources MP contamination within Table Bay including Salt River, Liesbeek River, Black River, Elsieskraal River, Camps Bay stream, Diepsloot River, Blinkwater River, Kasteelpoort River, Platpoort River and Lekkerwater River [23]. Studies have identified the Black River as a source of pollution due to effluents from industrial and residential areas in and around Cape Town [22]. The coastal zone is a mixed-use area where commercial, residential and recreational activities take place. A notable feature of this region is a WWTW dedicated to processing domestic waste. Numerous stormwater outfall pipes discharge directly into the nearshore environment and contaminate coastal surface waters [29].

False Bay is located on the south side of the Cape Peninsula, southeast of the Table Bay region. The bay extends from Cape Hangklip on the east to Cape Point on the west [30]. Near the mouth of the bay, surface currents flow in a westward direction and are controlled by weather, shelf waves, the warm Agulhas rings and eddies [31]. During summer, southeasterly winds cause offshore transport and upwelling at Cape Hangklip, which enters False Bay. There are 11 small estuaries and river systems that discharge directly into False Bay [32] including the Sand River, Zeekoei River, Lourens River, Sir Lowry’s River and the Steenbras River. Pressures surrounding the rivers and estuaries within False Bay are a result of highly urbanised areas, WWTWs, industrial effluent, urban and agricultural runoff from stormwater systems, degraded catchments, development of marinas, harbours, mouth manipulation and recreational activities [30,33,34].

### 2.2 Field sampling

Sampling for water and sediment took place in February (summer) 2020 at 14 sites in the Western Cape, South Africa. Sites 1 to 4 were in the West Coast region, sites 5 to 7 in the Table Bay region and sites 6–14 in the False Bay region (Fig. 1). Sites were chosen based on urban/rural anthropogenic influence, distance to sources of water (stormwater outfall pipes, WWTWs, river mouth) and harbours (Table 1). Sites 1 and 2 were located 150 m from a closed river mouth. Site 3 was located within in the Berg River. Sites 4, 5, 6, and 7 was located close to stormwater outfall pipes. Site 8 was located within Simon’s Town marina and sites 9 and 11 was located within 100 m of a stormwater outfall pipe. Site 10 was located 100 m from a WWTW. Site 12 was located 100 m outside a harbour. Sites 13 and 4 was located within 200 m from an open river mouth (Rooi Els River and Buffels River respectively). Site 14 was the least impacted by anthropogenic activities and considered a reference/control site.

**Table 1:** Site locations and descriptions of sites sampled.

Site no.	Region	Site Name	Site type	Co-ordinates (Longitude/Latitude)	Population	Urban/Rural	Potential MP sources	Distance to nearest water source (m)^a^
1	West Coast	Lambert’s Bay	Rocky Shore	18.313–32.085	6120	Rural	River (closed)	150
2	West Coast	Eland’s Bay	Rocky Shore	18.335–32.316	1525	Rural	River (closed)	150
3	West Coast	Velddrif	Harbour	18.143–32.771	11 017	Rural	River	0
4	West Coast	Saldanha Bay	Rocky Shore	17.946–33.007	111 173	Urban	Outfall pipe	80
5	Table Bay	Blouberg	Rocky Shore	18.463–33.805	172 601	Urban	Outfall pipe	30
6	Table Bay	Mouille Point	Rocky Shore	18.364–33.899	253 301	Urban	Outfall pipe	10
7	Table Bay	Maiden’s Cove	Rocky Shore	18.374–33.945	253 301	Urban	Outfall pipe	30
8	False Bay	Simon’s Town	Marina	18.430–34.190	6700	Urban	Marina	0
9	False Bay	Kalk Bay	Rocky Shore	18.450–34.127	700	Urban	SWOP^b^	10
10	False Bay	Strandfontein	Rocky Shore	18.553–34.089	37 911	Urban	WWTW	100
11	False Bay	Strand	Rocky Shore	18.825–34.117	55 558	Urban	SWOP	50
12	False Bay	Gordan’s Bay	Rocky Shore	18.858–34.166	16 776	Urban	Harbour	100
13	False Bay	Rooi Els	Rocky Shore	18.820–34.298	125	Rural	River (open)	200
14	False Bay	Pringle Bay	Rocky Shore	18.829–34.343	801	Rural	River (open)	5

a Outfall pipe, stormwater outfall pipe, river mouth, harbour/marina and wastewater treatment facility.

b Stormwater outfall pipes.

### 2.3 Water sampling and microplastic analysis

Five replicate 20 L of water were collected using a metal bucket per site. Water samples were filtered on site with a 250 μm metal sieve where the remaining particles on the mesh were transferred to a (pre-cleaned) Falcon tube, stored on ice and later stored in freezer until further analysis. Collection took place downwind of the sampling area to minimise MP contamination from clothes worn. Water samples were collected and processed based on the method by Ref. [24]. All glassware were autoclaved before use to minimise contamination. Water samples were removed from the freezer and left to thaw at room temperature. Samples were then transferred to a pre-cleaned glass jar and digested in 10% potassium hydroxide (KOH) (w/v, Merck, South Africa) (ratio of 1:2) for 24 h at 50 °C. To make 1 L solution of 10% KOH, 100 g of KOH was added to 900 ml of filtered reverse osmosis (RO) water and stored in a dark bottle. Samples were then filtered through a 20 μm (47 mm, Dawning Manufacturing, South Africa) mesh using a Buchner Funnel system and vacuum pump. The jar was rinsed 3 times with 10 μm filtered RO water and filtered through same 20 μm mesh. The mesh was placed in a petri-dish to prevent and minimise airborne contamination. The samples were then air-dried before analysing for MPs under a stereo microscope.

### 2.4 Sediment sampling and microplastic analysis

Sediment samples was collected at the high tide strandline parallel to the coastline. Collection took place downwind of the sampling area to minimise MP contamination. Five random replicates (5 m apart) along the strandline were sampled at each site using a 0.25 m × 0.25 m quadrate and sediment collected at a depth of 5 cm using a metal spoon. Sediment samples were stored in Ziploc bags and stored until further analysis. In the lab, sediment samples were removed from the Ziploc bags and separated into 2 (sample A and sample B) aluminium containers for each site. Sample A was used for MP analysis and sample B was used for grain size analysis. Samples were then covered with aluminium foil and placed in the oven to dry at 60 °C (24 h). Once dried, 100 g of dry sediment from sample A (MP analysis) was placed into a glass jar and digested in 10% KOH (ratio of 1:2) for 24 h at 50 °C. A hypersaline solution using NaCl (Technical grade, Science World, South Africa) was prepared by adding 360 g NaCl to 1 L filtered RO water. The hypersaline solution was filtered through a 10 μm mesh to remove potential contaminants in the NaCl. To extract MPs from sediment samples, the hypersaline solution was added to digested sediment sample (ratio 1:2 sediment:hypersaline solution) and stirred vigorously with a metal spoon for 2 min. The sample was then left to settle for 15 min. Once the sample was settled, the supernatant was filtered through a 20 μm mesh using a Buchner funnel system and vacuum pump. This process was repeated 3 times for the same sediment sample. The mesh was placed in a petri-dish to prevent and minimise airborne contamination. The samples were then air-dried before analysing for MPs under a stereo microscope.

To conduct grain size analysis, 150 g of dry sediment of sample B was sieved through 1180 μm, 500 μm, 250 μm, 125 μm, 63 μm and <63 μm mesh using a sediment shaker for 5 min. Sieves were then weighed and the data processed with GRADISTATv9.1 to measure the ratios of respective sediment size classes.

### 2.5 Microplastic visual identification and FTIR analyses

Identifying and recording MPs was done according to methods of [6]. Samples were visually sorted under a Zeis DV 4 dissecting microscope (x30) and MPs were categorised according to shape (filament, film, fragment, sphere), color (white, transparent, yellow, red/pink, blue/green, black/grey) and size (<100 μm, 100–500 μm, 500–1000 μm, 1000–2000 μm, 2000–5000 μm, >5000 μm). The size category was measured using 1 × 1 mm graph paper placed at the bottom of petri-dish. MP particles were identified by possessing unnatural shape/type, coloration and size. MP counts were peer reviewed to ensure all MP are accounted for. Once identified, 10% of putative MPs (>500 μm) were identified according to their polymer type using spectroscopy (PerkinElmer Two ATR-FTIR spectrometer) following the methods of [35]. Spectral wave numbers ranged from 4000 to 450 cm-^1^, resolution set to 4 cm^-1^, data interval set to 1 cm^-1^ and scans set to 10. A background scan was done before starting FTIR scans and the ATR crystal was cleaned with propanol between scans. Polymer identification was done by comparing spectral scans with the ST Japan Library and a Perkin spectral library provided by the supplier (PerkinElmer).

### 2.6 Quality control, method validation and limitations

To minimise plastic contamination within field sampling and laboratory analysis, the use of plastic was minimised in favour of metal and glass equipment and instruments. Lab doors and windows were closed to minimise airborne contamination and all work benches cleaned with ethanol before starting any processing of samples. Metal and glassware were autoclaved, then rinsed with MilliQ ultra-pure water before use. The same clothes were worn so that it was easy to identify and eliminate MPs from samples collected in the field and when processing samples in the lab. Water, 10% KOH and hypersaline solutions were filtered through 20 μm mesh to minimise MP contamination. Samples and solutions in lab were covered with foil to prevent and minimise airborne contamination. As a validation step, extraction efficiencies were done by filtering a known number of filaments and processed the same as water processing procedure. MPs were then counted and recovery percentage was calculated (at least 80% was recovered). Blanks (negative controls) were filtered to eliminate possible contamination from the filtration system. Positive controls (petri-dish with damp filter paper) were placed at the processing station in the lab to capture airborne contamination for the duration of lab work. The controls are checked at the start and end of each day and contamination was recorded. Recorded contamination was then subtracted from analysed samples.

The limits of detection for this study was 20 μm for the smallest MPs extracted as we used a 20 μm mesh. All MPs larger than 5 mm were not included in the results. For FTIR analyses, we only removed putative MPs larger than 500 μm to identify polymer type.

### 2.7 Risk assessment

Microplastic indices were applied to all samples to assess the potential risks of MPs in the environment, based on risk categories. The concentration of MPs (C_microplastic_) compared to background concentrations was assessed using a MPs contamination factor (MPCF)

(1) $$MPCF_{i}=\left(\frac{C_{microplastic}}{C_{baseline}}\right)$$

where the C_baseline_ value is the lowest average number of MPs at a particular site for water (Rooi Els) and sediment (Simon’s Town) samples, as there is no available historical data for the area and this method is considered acceptable [36]. MP pollution load index (MPPLI) was calculated as follows

(2) $${MPPLI}_{site}=\sqrt[{2}]{MPCFil\times MPCFilm}$$

where MPCFil and MPCFilm were MPCFs for filaments and film, respectively. Filament and film particles were the most abundant MP type across all samples types. The chemical toxicity of polymers was analysed based on the method used by Ref. [25], where hazard scores are assigned to polymer types to assess the risk of polymers

(3) $$H_{i}=\sum P_{n}\;X\;S_{n}$$

where H_i_ is the calculated polymer risk index, P_n_ is the ratio of a polymer type recorded and S_n_ is the polymer hazard score assigned by Ref. [25]. The pollution risk index (PRI) was calculated as follows

(4) $$PRI=\Sigma \;H_{i}\times {MPPLI}_{site}$$

where PRI_i_ is the ecological hazard of polymers associated with polymer risk index (H_i_).

Once the respective risk indices were calculated, the scores were assigned to categories based on the severity of the potential risk, ranging from low risk (I) to dangerous (V) (Table 2).

**Table 2:** Risk categories of indices for microplastic contamination in samples collected along the Western Cape coastline, South Africa.

Risk Category	Low (I)	Moderate (II)	High (III)	Very high (IV)	Dangerous (V)
Contamination Factor (CF)	<1	1–3	3–6	>6	
Pollution Load Index (PLI)	<1	1–3	3–4	4–5	>5
Polymer Risk Index (H)	<10	10–100	101–1000	1000–10 000	>10 000
Pollution Risk Index (PRI)	<150	150–300	300–600	600–1200	>1200

### 2.8 Data analyses

All statistical analysis was performed using IBM SPSS Statistics v28. MP data was expressed as MP counts per L (particles/L) for water and dry weight per kg (particles/Kg) for sediment. Descriptive statistics was calculated for sample type (water, and sediment), region, urban/rural and site. Data was tested for normality using the Kolmogorov-Smirnov test in conjunction with reviewing the skewness and kurtosis values and histograms. Data were not normally distributed and non-parametric analyses conducted using the Kruskal-Wallis H tests for analysing significant differences and H represents degrees of freedom between more than 2 groups and a Mann-Whitney *U* test for two groups (urban/rural) for different sample types (water and sediment). Post-hoc tests with pairwise comparisons using the Kruskal-Wallis H method were conducted to show significant different between categories (the significance level was set at p < 0.05). Variance of data for statistical analysis was presented using standard error of the mean (SE). Spearman rank correlations were done to determine the relationship between variables.

## 3 Results

### 3.1 MP concentrations and distribution

**Fig. 2:**
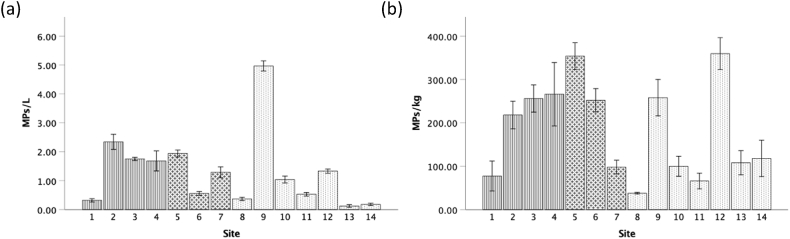
Microplastic concentration in a) water (particles/L) and b) sediment (particles/kg) samples collected at various sites along the Western Cape coastline. Sites 1–4: West Coast, 5–7: Table Bay and 8–14: False Bay.

Of the 70 water samples processed, 69 (99%) contained MP particles. A total of 1840 MP particles were recorded in water samples (mean of 1.33 ± 0.15 SE particles/L) (Fig. 2a). At a regional scale, mean water MP concentration was highest in the West Coast region (1.52 ± 0.20 particles/L). There was a significant difference between MP concentrations in water samples across the regions (H (2) = 7.376, p = 0.025), with the West Coast being significantly higher than False Bay (H (2) = −2.493, p = 0.013). The mean MP concentration in urban areas were significantly higher (1.52 ± 0.20 particles/L) than rural areas (0.98 ± 0.20 particles/L) (U = 705, p = 0.038). Water MP samples were significantly highest (H (13) = 62.891, p < 0.01) in Kalk Bay (site 9) (4.97 ± 0.18 particles/L) and lowest at Rooi Els (site 13) (0.13 ± 0.05 particles/L).

For sediment samples (N = 70), 69 (99%) contained MP particles. A total of 1277 particles were recorded, with a mean concentration of 185.07 ± 15.25 SE particles/kg (Fig. 2b). At a regional scale, highest mean MPs in sediment concentrations were in Table Bay (234.67 ± 31.42 particles/kg) and lowest along the West Coast (211.05 ± 27.05 particles/kg) and False Bay (149.71 ± 21.17 particles/kg). There was a significant difference between MP concentrations in sediment samples across the regions (H (2) = 6.646, p = 0.036), with the pairwise comparison showing a significant difference between Table Bay and False Bay (H (2) = −2.273, p = 0.023). The mean MP concentration in urban areas (199.11 ± 20.69 particles/kg) were significantly higher when compared to rural areas (158.75 ± 19.83 particles/kg) (U = 614.500, p = 0.348). The mean MP sediment concentrations varied across sites, with Gordan’s Bay (site 12) displaying the highest concentration (360.00 ± 36.74 particles/kg) and Simon’s Town the lowest (38.00 ± 2.00 particles/kg).

### 3.2 Grain size analysis and correlations

**Fig. 3:**
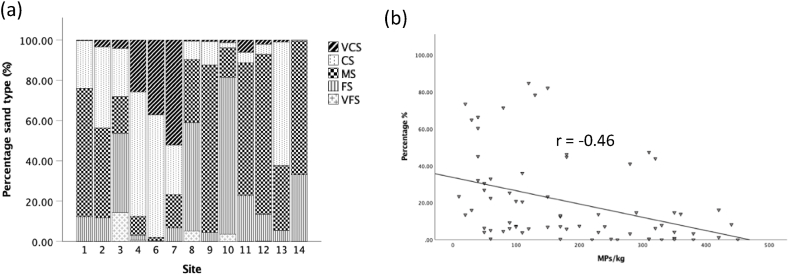
a) Sediment type percentage of samples collected at each site along Western Cape coastline, South Africa and b) correlations between MPs and fine sand. VCS: Very Coarse Sand (1–2 mm); CS: Coarse Sand (500–1000 μm); MS: Medium Sand (250–500 μm); FS: Fine Sand (125–250 μm); VFS: Very Fine Sand (63–125 μm). Note Blouberg (site 5) sediment was not classified due insufficient data.

Grain size analysis was conducted to determine grain size distribution at sampling sites using GRADISTATv9.1 (Fig. 3a). All sediment was classified as sand, with coarse sand (1180–500 μm) and medium sand (500–250 μm) being the most dominant sediment type for all sites. Coarse sand (CS) was the most dominant grain size in Saldanha Bay (site 4), Rooi Els (site 13) and Eland’s Bay (site 2) (62%, 61% and 40% respectively), whereas medium sand (MS) was the most dominant grain size in Kalk Bay (site 9), Gordan’s Bay (site 12), Strand (site 11) and Lambert’s Bay (site 1) (83%, 79%, 66%, 66% and 64% respectively). There was a noticeably high amount of fine sand (FS) in Strandfontein (site 10) and Simon’s Town (site 8) (77% and 97% respectively).

Spearman rank correlations showed no relationship (r = < 0.1) between MP concentrations and grain size for all grain sizes analysed, except for fine sand (FS) showing a weak inversely proportional relationship with significant difference (r = - 0.46; p = 0.001) (Fig. 3b).

**Fig. 4:**
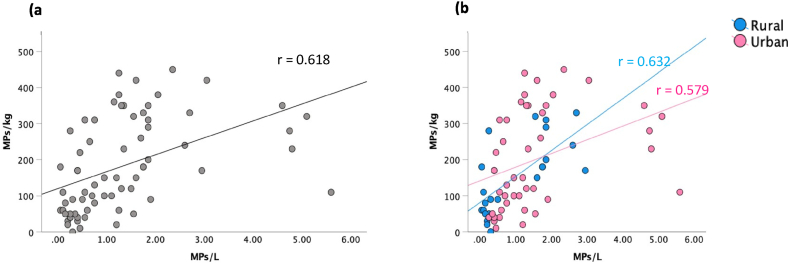
a) Correlations between microplastic concentrations in water (particles/L) and sediment (particles/kg) and b) correlations in urban and rural areas samples collected along the Western Cape coastline, South Africa.

There was a strong (positive) correlation (r = 0.62; p < 0.001) between MP concentrations in water and sediment samples collected along the coastline of the Western Cape, South Africa (Fig. 4a). There was also a strong positive correlation between MP concentrations in water and sediment samples collected from rural areas (r = 0.632; p < 0.001) compared to urban areas (r = 0.579; p < 0.001) (Fig. 4b). The results show a directly proportional relationship between MP concentrations in water and sediment samples except for a few outliers.

### 3.3 MP shape, color and size

**Fig. 5:**
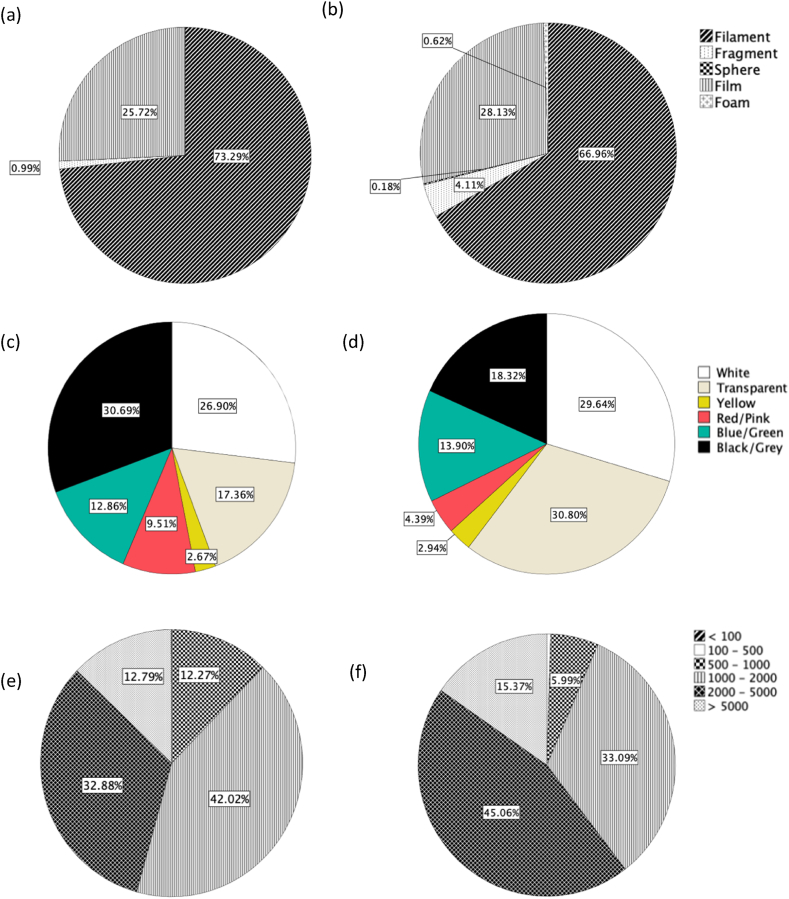
Percentage of microplastic shape in a) water and b) sediment samples combined; microplastic color in c) water and d) sediment samples combined and microplastic size in e) water and f) sediment samples combined.

The most dominant MP shape recorded in all water samples (Fig. 5a) were filaments and film (73% and 26% respectively), where fragments were the least abundant MP shape (1%). At a regional scale, filaments were most prevalent in Table Bay (80%), whereas film was highest along the West Coast (36%). The most dominant MP shapes recorded in water samples from urban and rural areas were filaments (75%) and film (29%). MP shape varied in water samples across sites, with the most dominant type being filaments at Simon’s Town (site 8) (98%) and film at Saldanha Bay (55%). MP shape varied in sediment samples across regions, urban and rural areas and sites (Fig. 5b). The most dominant MP shape recorded in all sediment samples was filaments and film (67% and 28% respectively). At a regional scale the percentage of filaments and film was highest at Table Bay (72%) and along the West Coast (37%). The most dominant MP shapes in rural and urban areas were filament (68%) and film (29%). MP shape varied in sediment samples across sites, with the most dominant shape being filament at Kalk Bay (98%) and film at Saldanha Bay (57%).

The dominant MP colors recorded in water samples were black/grey, white and transparent (31%, 17% and 17% respectively) (Fig. 5c). At a regional scale, black/grey MPs were highest in False Bay (37%) and white and transparent highest along the West Coast (39%) and in Table Bay (23%). White, transparent and black/grey MPs were highest in rural areas (33%, 18% and 32% respectively), whereas blue/green MPs were highest in urban areas (17%). MP colors varied in water samples across sites and white MPs were highest at Blouberg (site 5) (53%) and Maiden’s Cove (site 7) (38%), with red/pink and black/grey MPs highest at Simon’s Town (site 8) (53%) and Pringle Bay (site 14) (59%). The most dominant MP color recorded in all sediment samples (Fig. 5d) were white, transparent, blue/green and black/grey (30%, 31%, 14% and 18% respectively). At a regional scale, white and transparent MPs were highest along the West Coast (37% and 33%), whereas blue/green and black/grey MP particles was highest in False Bay (17% and 22%). Transparent, blue/green and black/grey MPs were highest in rural areas (33%, 14% and 19% respectively), whereas white MP particles were highest in urban areas (21%). MP colors varied in sediment samples across sites, with the most dominant colors being white, transparent, blue/green and black/grey. White and transparent MPs was highest at Saldanha Bay (site 4) (57%) and Velddrif (site 3) (57%), whereas blue/green and black/grey MPs were highest at Simon’s Town (site 8) (35%) and Kalk Bay (site 9) (35%).

MP sizes varied in water samples across regions, urban/rural areas and sites along the Western Cape coastline (Fig. 5e). The most dominant MP sizes recorded in water samples were 1000–2000 μm (42%) and 2000–5000 μm (33%). At a regional scale, MP particles 1000–2000 μm and 2000–5000 μm were highest along the West Coast (48%) and in Table Bay (48%). The most dominant MP sizes in rural and urban areas were 1000–2000 μm (50%) and 2000–5000 μm (36%). The sizes of MPs varied in water samples across sites, with the most dominant size being 1000–2000 μm, 2000–5000 μm and >5000 μm in samples collected from Lambert’s Bay (site 1) (75%), Eland’s Bay (53%) and Kalk Bay (site 9) (63%). MP size varied in sediment samples across regions, urban and rural areas and sites along the Western Cape coastline (Fig. 5f). The most dominant MP sizes recorded in all sediment samples were 1000–2000 μm (33%) and 2000–5000 μm (45%). At a regional scale, the percentage of MP particles 2000–5000 μm were highest along the West Coast (41%) and in False Bay (49%). The most dominant MP sizes in rural and urban areas were 1000–2000 μm (34%) and 2000–5000 μm (46%). The size of MPs varied in sediment samples across sites, with the dominant sizes being 2000–5000 μm in samples collected from Velddrif (site 3) (58%) and Strand (site 11) (39%).

### 3.4 Polymer identification

**Fig. 6:**
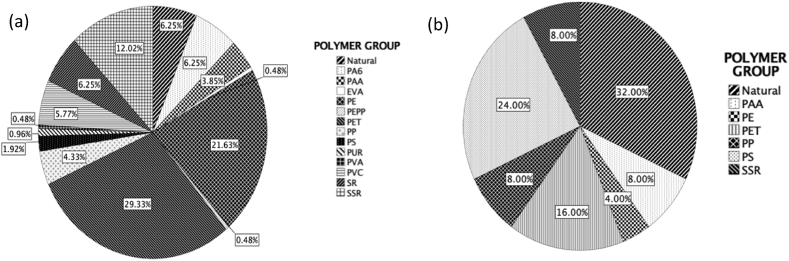
Polymer identification of overall MPs in a) water and b) sediment samples collected along the Western Cape coastline, South Africa. Natural: Cellulose/protein based particles; EVA: Ethylene vinyl acetate; PA6: Polyamide 6; PAA: Polyacrylic acid; PE: Polyethylene; PEPP: Polyethylene propylene; PET: Polyethylene terephthalate; PP: Polypropylene; PS: Polystyrene; PUR: Polyurethane; PVA: Poly vinyl acetate; PVC: Poly vinyl chloride; SR: Synthetic rubber; SSR: Semi-synthetic rubber.

A total of 10% of particles identified as putative MPs were processed for FTIR analysis to ascertain polymer types. For all water samples (Fig. 6a), polyethylene terephthalate (PET) and polyethylene (PE) were the most abundant polymer types (29% and 22% respectively). Polymers varied with MP type, with PET and PE being the most abundant polymer type in filaments (42%) and film (54%). The most abundant polymer type of foam and fragment particles were polystyrene (PS) (100%) and polyacrylic acid (PAA) (50%). False Bay displayed the most variability in polymer type, with PET and PE being the most abundant polymer type (29% and 25% respectively). The West Coast region displayed the least variability in polymer type but had the highest percentage of semi-synthetic rubber (SSR) (39%). Urban areas displayed the most variability in polymer type, with PET and PE being the most abundant (28.57% and 23.81% respectively). Rural areas were mainly PET (37%) and SSR (26%).

Natural filaments (32%), PS (24%) and PET (16%) were the most prevalent MP polymer categories in sediment samples (Fig. 6b). The dominant polymer type of film and foam particles were identified as PE (667%) and PS (100%), respectively. The West Coast had the highest variability in polymer type, with Natural (39%) and PS (22%) being the most dominant categories. In False Bay, PET (50%) was most prevalent and in Table Bay, PS (40%) most common. Rural areas had the highest percentage of Natural, PAA and PET (46%, 18% and 18% respectively), whereas urban areas were mainly PS and SSR (36% and 14% respectively).

### 3.5 Risk assessment of microplastics in water and sediment

**Fig. 7:**
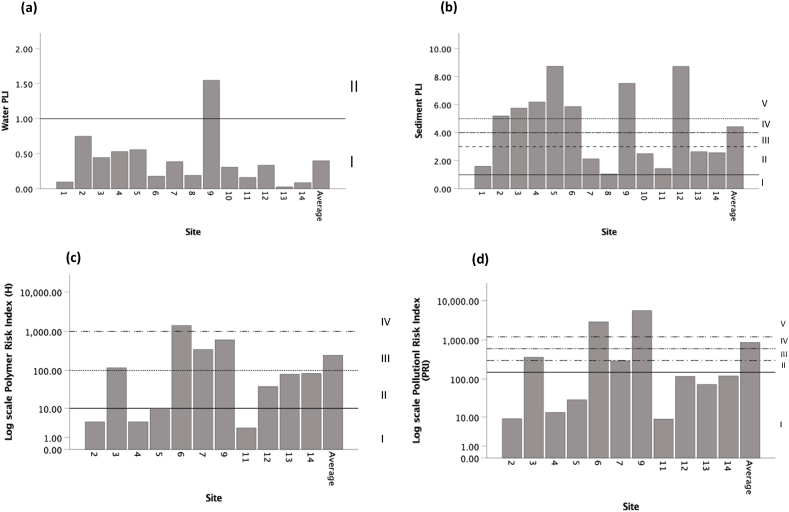
Pollution Load Index (PLI) in a) water and b) sediment samples. Log Polymer Risk Index (H) c) and log Pollution Risk Index (PRI) d) of microplastics in all samples at each site along the Western Cape coastline, South Africa. Note the log scale for both polymer risk index (H) and pollution risk index (PRI). See Table 2 for categories of indices.

The MP Pollution Load Index (PLI) indicated low pollution loads in water samples collected at all sites (Fig. 7a), except for Kalk Bay (site 9) where a moderate pollution load (1.55) was recorded. The MP PLI in sediment samples (Fig. 7b) varied across all sites, with dangerous pollution loads (category V) at Eland’s Bay (site 2) (5.20), Velddrif (site 3) (5.75), Saldanha Bay (site 4) (6.20), Blouberg (site 5) (8.75), Mouille Point (site 6) (5.87), Kalk Bay (site 9) (7.53) and Gordan’s Bay (site 12) (8.73). The Polymer Risk Index (H) was categorised as dangerous at Mouille Point (site 6) (1399) and very high at Velddriff (site 3), Maiden’s Cove (site 7) and Kalk Bay (site 9) (Fig. 7c). The Pollution Risk Index (PRI) (ecological risks) were generally categorised as low to moderate risk. High PRI values were recorded at Velddrif (site 3) and dangerous at Mouille Point (site 6) (2909) and Kalk Bay (site 9) (5579) (Fig. 7d).

## 4 Discussion

### 4.1 Water and sediment MP concentration and distribution

Based on region, MPs were highest along the West Coast of the Western Cape (Fig. 1, Fig. 2a), an area that is predominantly rural with agricultural activities. The relatively highest concentrations may be linked to informal settlements using water systems (mainly rivers) to wash clothes and some settlements discarding domestics waste into river and stormwater systems. This postulation is supported by Ref. [37] who reported that filaments were highest in coastal rural areas in South Africa where residents did not have access to piped water. The higher records of filaments were based on the postulation that rural communities in South Africa rely on rivers as their primary source of water for washing clothes. Other factors that may have contributed to MPs in water along the West Coast include stormwater outfalls [15,29], WWTW discharge points [29,38,39] and harbours [16,39]. The significantly higher water MP concentrations at Kalk Bay could be related to the site being in a sheltered area with weak water circulation [34], within 10 m of a stormwater pipe, a popular tourist destination and a popular fishing spot.

MP concentrations were highest in sediment samples collected in Table Bay (Fig. 2b). Table Bay is a shallow bay and surface current flows generally weak [28] that may be enabling MP particles to settle in sediment. Table Bay forms part of Cape Town harbour where reports of WWTW spills and discharges from harbour activities have been linked to marine pollution. Other potential sources of MP contamination could be linked to river systems (Salt River, Liesbeek River and the Black River) directly entering Table Bay [29]. The Black River has been identified as a source of marine pollution due to effluent from industrial and residential areas along its catchment. Another notable feature about this region is a WWTWs, discharging effluent directly into nearshore environments, which have been identified as sources of MP contamination [29]. Sediment MPs were highest at Gordon’s Bay (site 12), where samples were collected in a semi-enclosed area and 100 m from a harbour. Research has shown MP concentrations being linked to harbour and maritime-related activities [16,19,40]. In addition, semi-enclosed shallow areas have weak water circulation, allowing MPs to settle in sediment.

Water MP concentrations recorded in our study are similar to other sites in South Africa (Durban) and globally (Sweden and Australia), but lower when compared to harbors in South Africa (Durban and Richards Bay) (Table 3). Sediment MP concentrations were comparable to other global sites (Belgium and Germany), but lower when compared to other African countries (Tanzania) and elsewhere in South Africa (south-east coastline).

**Table 3:** Comparison of coastal water and sediment MP concentrations with other investigations.

Sample type	Location	Site description	Average (or range) MP Concentrations	Reference
**Water** (Particles/L)	Western Cape, South Africa	Rocky shores	1.33	*This study*
	SE coastline, South Africa	Coastal waters	(258–1215)	[40]
	Durban, South Africa	Harbour	1200	[39]
	Richards Bay, South Africa	Harbour	413	[39]
	Sweden	Harbour	(0.15–2.40)	[41]
	Australia	Harbour	(0.06–2.50)	[42]
**Sediment** (Particles/kg)	Western Cape, South Africa	Rocky shores	185	*This study*
	Simon’s Town	Marina	5769 (median)	[24]
	South Africa	Beaches	83.5 (fibres only)	[29]
	SE coastline, South Africa	Coastal waters	(688–3308)	[40]
	Tanzania	Beaches	2972	[43]
	Belgium	Intertidal	92	[44]
	Germany	Beach	106	[45]

### 4.2 MP shape, color and size

Similar predominant MP shapes (filaments) were recorded for water and sediment samples. Various studies have reported filament as the most dominant MP type in coastal areas and Cape Town [29,37,46]. This is because filaments behave differently in the ocean due to their surface area to mass ratio being greater than other MP types [47]. In addition, biofouling rates are much higher on these MP types than on other MP types, causing filaments to sink sooner over a shorter period. Once on the beach, filaments are incorporated into the sediment matrix through pore-migration [48]. Filaments are more susceptible to being trapped within sediment [49] and this could explain the relatively high abundance of this MP type in the sediment. The color and size variability recorded could be a result of secondary MPs being formed by a combination of photo-degradation, mechanical transformation from wave action and biological degradation by organisms [3,13,50]. MP size is important for understanding how MPs are transported and spatially distributed in the marine environment via currents and waves relative to their hydraulic equivalence to natural sediment particles [48,50]. Given that most MPs were filaments, this could explain why MP particles in this study ranges were mainly 1000–2000 μm and 2000–5000 μm.

### 4.3 Correlations

The correlations reported displays the way MPs interact and reside within the marine environment. There was a strong correlation between MP concentrations in water (particles/L) and sediment (particles/kg). This implies that MPs present in the water column eventually settle in marine sediment [49]. However, it is important to note that where MP concentrations were higher in water samples in relation to sediment samples, it could indicate MPs were being resuspended through bioturbation by organisms, wave action, upwelling events, change in physical characteristics such as size, shape and density, ageing and weathering of MPs [51].

The results showed a weak correlation between sediment grain size categories with significant correlations only recorded between MP concentrations and fine sand (r = −0.391; p = 0.001). This suggests that MP concentrations are affected by sediment properties [52]. The hydraulic equivalence of plastic particles of a particular shape, size and density behave like naturally occurring sediment particles of similar shape, size and density in the environment [48,53]. The lack of correlations between MPs and other grain sizes could be linked to environmental conditions of the location along the Western Cape coastline, in particular potential sources, particle size, shape and density. The Western Cape coastline has a relatively narrow shelf and the hydrodynamics along the coastline is affected by the direct interaction between land and sea and associated air masses [27,28]. Studies have found that the shape, width and circulation along coastlines affect hydrodynamic forces, which are factors that influence deposition of MPs along shorelines [52].

### 4.4 Polymer identification

For all the sites combined, the most dominant polymer types recorded in water samples were PET and PE. PET applications include the textile industry which are susceptible to oxygen and UV degradation [54] and could explain the high abundance of PET filamants in the marine environment. PET has a high density and settling rate. However, these particles have the ability to be resuspended in the water column through bioturbation by organisms, upwelling events, change in physical characteristics, such as size, shape and density, ageing and weathering [51]. PE is used to produce plastic film and applications include carrier bags, cling wrap and freezer bags [54]. False Bay displayed the highest variability in polymer type followed by Table Bay and both regions are subject to large scale winds [27], allowing plastics from different areas to be blown into the ocean. In addition, there are various products linked to sources that could potentially explain the variation in polymer types, ranging from industrial areas, WWTWs, river runoff, to harbour and fishing related activities.

For all sediment samples combined, the most dominant polymer types recorded was Natural (32%), followed by PS (24%) and PET (16%). The West Coast region had the highest variability of polymer types, with Table Bay having the highest percentage of PS (40%). The West Coast region is a mixed-used area with potential sources of MPs from activities such as fishing, stormwater outfalls, river input, agriculture to harbour and aquaculture. Products emanating from sources could explain the variations in polymer type recorded. The Table Bay region forms part of Cape Town harbour and has weak water circulation. As a result, low density PS based plastics can be susceptible to biofouling, making plastic denser causing it to sink and settle in sediment. Even though PS is generally water resistant, it can absorb water once it comes into direct contact, increasing its density and facilitating in it sinking.

### 4.5 Risk assessment

MPs are comprised of numerous polymers produced by polymerization of monomers and the addition of hazardous additives [25,55]. Most plastics are categorised as hazardous according to a model from the United Nations’ Globally Harmonized System of Classification and Labelling of Chemicals [25,55,56]. Hence, the consumption of MPs poses health and ecological risks [56] and the ecological risk assessments conducted in this study attempts to indicate what risks do MPs from coastal water and sediment in the Western Cape of South Africa pose. The Pollution Load Index (PLI) was categorised as generally low for water samples and very high in sediment samples across all the sites. These high concentration levels have the potential to threaten marine species inhabiting the sediment. In addition, it has potential to be resuspended through wave action and bioturbulance, which are factors that reintroduce MPs into the water column and could be ingested by marine organisms. The Pollution Risk Index (H) and Polymer Risk Index (PRI) for all samples combined were generally very high and dangerously high at Mouille Point (site 6). Mouille Point did not necessarily display the highest MP concentration but had polymers with high hazard scores (PUR and ABS) [25]. This indicates that the risk MPs poses on the environment is associated with the polymer type and not necessarily the MP concentration. This is important to note as the effects associated with polymer types could potentially pose a threat to marine organisms ingesting MPs.

## 5 Conclusion

This study provides data of MP concentrations and potential sources in water and sediment at various sites along the Western Cape coastline. Results suggest spatial distribution in water and sediment varies with sources and environmental conditions along the coastline. One major source of contamination is due to stormwater outfall pipes, with filamentous particles being the dominant MP type. The risk assessment indicates high to dangerous risks associated with MP polymer type and not necessarily MP concentration. This is important to note as the effects associated with polymer types could potentially pose a threat to marine organisms ingesting MPs.

## Author contribution statement

Danielle Julius: Performed the experiments; Analysed and interpreted the data; Wrote the paper.

Adetunji Awe: Analysed and interpreted the data; Contributed reagents, materials, analysis tools or data.

Conrad Sparks: Conceived and designed the experiments; Analysed and interpreted the data; Contributed reagents, materials, analysis tools or data; Wrote the paper.

## Declaration of competing interest

The authors declare that they have no known competing financial interests or personal relationships that could have appeared to influence the work reported in this paper.
